# Evaluation of a Collaborative Care Management Program for Community-Dwelling Older Adults on High-Dose Opiates

**DOI:** 10.1093/geroni/igaa057.668

**Published:** 2020-12-16

**Authors:** Shahrzad Mavandadi, Kristin Foust-Montague, Elizabeth Grecco, David Oslin, Joel Streim

**Affiliations:** 1 Corporal Michael J Crescenz VA Medical Center, Aliso Viejo, California, United States; 2 Perelman School of Medicine at the University of Pennsylvania, Philadelphia, Pennsylvania, United States; 3 Perelman School of Medicine at the University of Pennsylvania, Philadelphia, United States

## Abstract

Treating pain in later life is complex, and there are significant safety risks associated with the use of analgesics, particularly opioids. This study examined preliminary results from a pilot study of a telephone-delivered collaborative care service designed for community-dwelling older adults with chronic pain receiving prescriptions for high doses of opioids (i.e., >120 mg morphine-equivalent dose). Eighty-two older adults referred by the Pennsylvania Department of Aging’s pharmaceutical assistance program for low-income seniors (PACE/PACENET) were eligible and enrolled in the program (i.e., the University of Pennsylvania/PACE Behavioral Health Laboratory). Patients were on average 73.5 (+/-6.1) years old, and the majority were white (91%) and female (70%). Patients completed a comprehensive baseline clinical assessment capturing their mental health, cognition, pain and functional status, as well as self-reported daily opioid dose and biopsychosocial needs. Patients were considered engaged in the program if they completed 2+ additional follow-up contacts with a care manager. During these contacts, care managers offered individualized treatment planning, with the goal of opioid dose reduction to safer levels. Of the 82 patients completing the baseline, 53 (65%) engaged in the program. At their last clinical contact, 91% of engaged patients achieved dose reductions (with 66% achieving dose reductions of >20% and 30% reporting doses <120 mg morphine-equivalent dose). Engaged patients also reported significant reductions in pain severity (p=0.05) and depressive symptoms (p=0.003) at the last contact relative to baseline. Findings support the feasibility of a community-based, collaborative care model for pain management and suggest the potential for positive treatment outcomes.

